# Task content of jobs and mothers’ employment transitions in Germany

**DOI:** 10.1186/s12651-024-00384-9

**Published:** 2024-12-12

**Authors:** Honorata Bogusz

**Affiliations:** Interdisciplinary Center for Labor Market and Family Dynamics (LabFam), University of Warsaw, Warsaw, Poland

**Keywords:** Task content of jobs, Employment transitions, Competing risk, Germany, J13, J16

## Abstract

I study the association between task content of jobs and mothers’ employment transitions after the first birth in Germany. I construct measures of task content of jobs using data from the Employment Survey conducted by the German Federal Institute for Vocational Education and Training (BiBB). These indicators illustrate the career cost of children and how it is impacted by the technology- and globalization-driven labour market change. The measures are then linked to high-quality individual register data from the German Pension Fund (FDZ-RV) covering the years 2012–2020. Utilizing competing risk models, I show that women engaged in occupations with analytic and interactive task content, which are in high demand and incompatible with maternity-related employment breaks, are the most likely to transition to employment after their first birth. Conversely, women with occupations intense in routine tasks, which are more susceptible to automation or trade competition, are more likely to experience unemployment.

## Introduction

Becoming a mother can considered a pivotal event in women’s careers. Indeed, numerous studies have identified maternity as a key contributor to gender inequality in Western societies (Kleven et al. 2019; Goldin 2021; Kleven et al. 2023). Women experience diverse employment trajectories following motherhood, with many opting for part-time work or choosing not to re-enter the workforce at all (Waldfogel et al. 1999; Arntz et al. 2017), particularly in conservative welfare regimes such as Germany (Gustafsson et al. 1996; Gutiérrez-Doménech 2005). Prior research has demonstrated that women with higher wages (Barrow 1999; Arntz et al. 2017), more secure job positions (Saurel-Cubizolles et al. 1999; Arntz et al. 2017), better education (Arntz et al. 2017), those from higher social strata (Saurel-Cubizolles et al. 1999), and those in professional jobs (Smeaton 2006) are more likely to re-enter employment after giving birth. Conversely, women from lower social strata, with lower educational attainment, and engaged in low-skilled occupations face the greatest risk of transitioning to unemployment post-maternity (Arntz et al. 2017). Existing research suggests, therefore, that maternity significantly exacerbates employment disparities among workers and impedes social mobility. This study contributes to the literature by examining the relationship between the task content of jobs and mothers’ return to the labour market-an exploration of the heterogeneous career costs associated with parenthood, which I argue may be exacerbated by the contemporary labour market shifts driven by technology and globalization.

Previous research on the career implications of parenthood has primarily focused on child penalties, referring to the sustained decrease in earnings or employment experienced by new mothers, a phenomenon not observed in men (instead, men often experience child premiums; see Baranowska-Rataj and Matysiak (2022)) or women without children. Across Western institutional contexts, child penalties are largely attributed to the reduction in hours worked by women following the birth of their first child (e.g., Kleven et al. 2019; Huber et al. 2023; Waszkiewicz and Bogusz 2023; Kleven et al. 2024). Some recent studies have suggested that cultural and gender norms underpin this gender disparity in labour market outcomes. Kleven et al. (2023) examined child penalties in employment across 134 countries worldwide and demonstrated that they constituted the largest component of gender inequality, with an increase in a country’s level of development and wealth. Andresen and Nix (2022) compared child penalties among different-gender couples with biological and adoptive children and among female same-gender couples (who generally exhibit less specialization than different-gender couples; see Ciscato et al. (2020)) in Norway. They found no disparity in penalties between different-gender biological and adoptive parents but identified considerably smaller and more evenly distributed penalties among female same-gender parents. The significance of norms concerning gender and parenthood was reinforced by Kleven et al. (2024), who demonstrated that family policies (such as the expansion of parental leave or childcare subsidies) had no significant impact on child penalties in Austria. Other studies conducted in German-speaking countries identified a positive effect of increased childcare availability on maternal part-time employment but observed no effect on women’s careers (Krapf et al. 2020; Huber et al. 2023).

While predominantly influenced by gender norms, the magnitude of child penalties in women’s earnings or employment is contingent upon their position on the career-family continuum and associated occupational choices, as demonstrated for Germany from 1972 to 2001 by Adda et al. (2017). Career-focused women in vocational training sorted themselves into occupations characterised by abstract (cognitive) tasks from an early age, while their family-oriented counterparts opted for occupations involving routine or manual work. Qualifications in jobs with abstract tasks are prone to evolve more rapidly than those in routine and manual jobs, necessitating constant skill updating-a demand that clashes with employment breaks associated with motherhood. Thus, a job intensive in abstract tasks might be less compatible with maternity than a job with routine or manual tasks. From this perspective, the task content of jobs can be viewed as reflecting the career costs of parenthood. Consistent with this argument, Adda et al. (2017) found that women in jobs involving abstract tasks were more inclined to remain childless or have only one child compared to their peers in routine and manual jobs. Furthermore, women in abstract jobs potentially face much higher opportunity costs of parenting due to steep earning profiles, a changing environment, and rapidly depreciating human capital, than women in routine or manual jobs. Consequently, women may be inclined to return to abstract jobs more quickly (in addition to being more career-oriented). However, the link between the task content of work and a woman’s return to the labour market after the first birth has not yet been addressed. Investigating this link is further motivated by the labour market transformations propelled by technology and globalisation, which took off in developed nations in the mid-20th century but gained momentum in the past three decades, thereby reshaping task demands (Acemoglu et al. 2011; Autor 2013; Lewandowski et al. 2022). As routine jobs increasingly become low-paid and unattractive, or disappear altogether, these shifts may have modified the extent to which tasks represent the career cost of children. They underscore the need for ongoing research into the task content of jobs and women’s labour market outcomes in more recent periods, as presented in this study.

Technological advancements and globalization have profound ramifications for the workforce (OECD 2019; World Bank 2019). Empirical economic literature suggests that automation and globalization contribute to significant polarization of job opportunities in Western labour markets, primarily through the deroutinization of work (Goos et al. 2009; Autor and Dorn 2013). Technology, skill supply, and globalization (in terms of trade liberalization) account for the majority of the contemporary shift from routine to non-routine cognitive work globally (Lewandowski et al. 2022). While the supply of skills (alongside technology) drives the transformation of work tasks for highly skilled professionals, globalization plays a more prominent role for workers in low-skilled occupations (Ibid.). Research indicates that automation and trade liberalization lead to decreased employment (Acemoglu and Restrepo 2020; Dauth et al. 2021; Keller and Utar 2023) and wages (Baumgarten et al. 2013; Acemoglu and Restrepo 2020), particularly affecting low- and middle-skilled workers, as well as those in the manufacturing sector, and exacerbating economic inequality across Western contexts (Huber and Winkler 2019; Doorley et al. 2023; Acemoglu and Johnson 2023). On one hand, lower-skilled workers face setbacks as their occupations (or specific tasks within occupations) are displaced by technologies such as industrial robots (Acemoglu and Restrepo 2020; Dauth et al. 2021) and Chat GPT (Eloundou et al. 2023; Felten et al. 2023; Gmyrek et al. 2023), or vanish due to increased import competition (Autor et al. 2013). Conversely, highly skilled professionals benefit, possessing analytical skills necessary for working with these technologies or skills integral to jobs involving human interactions, which are challenging for machines to replicate and cannot be outsourced (Deming 2017; Deming and Kahn 2018). Moreover, studies conducted in Europe have found that women are disproportionately represented in routine occupations, which are most susceptible to displacement and are increasingly of low quality (Piasna and Drahokoupil 2017; Brussevich et al. 2019).

This study is situated in Germany, arguably the most technologically advanced European country, where structural changes in the labour market are particularly pronounced, evidenced by the widespread adoption of industrial robots (Dauth et al. 2021; Deng et al. 2023) and increasing demand for cognitive labour (Spitz-Oener 2006; Rohrbach-Schmidt and Tiemann 2013; Bogusz et al. 2024). Germany is also one of the few European countries that maintain modernized manufacturing and compete in production processes (Dauth et al. 2017; Thelen 2019), rendering it potentially susceptible to import competition. Additionally, Germany is characterized by a conservative welfare regime, where many women transition to part-time employment upon becoming parents, and it is not uncommon for mothers, particularly in Western Germany, to exit the labour market for a longer period of time (Boll and Lagemann 2019; Mueller et al. 2020).

I measure the task content of jobs using the 2006 Employment Survey conducted by the German Federal Institute for Vocational Education and Training (BiBB) (Hall et al. 2006). These data enable me to construct five measures of task intensity commonly employed in economic literature (Autor et al. 2003; Spitz-Oener 2006; Hardy et al. 2018): analytic, interactive, non-routine manual, routine cognitive, and routine manual (with the latter two measures aggregated into a routine measure, as detailed in Sect. 2.2) at the three-digit occupation level. I link these occupation-specific measures to individual-level administrative data from the German Pension Fund for the years 2012 to 2020 (FDZ-RV 2024a, b). Employing the competing risk model (Fine et al. 1999), I explore women’s employment transitions following their first childbirth, with the task content of jobs serving as the primary covariate of interest. This model offers an advantage over standard duration models as it takes into account the possibility of individuals experiencing multiple events during the follow-up period. I distinguish between the following states that young mothers transition into after their maternity leave: employment, unemployment, and second birth. I focus on mothers’ return to the labour market and present supplementary findings for second birth in the appendix, complementing the main results for employment and unemployment. Transitions to other states, such as inactivity, present identification challenges (see Sect. 2.1). I do not explicitly examine them as outcomes, but treat them as censored cases. As women sort themselves selectively into specific occupations following their family-career orientation as early as in puberty (Adda et al. 2017), the results of the competing risk models presented here should be interpreted only as correlations.

The findings are consistent with previous labour market research in Germany, indicating that the likelihood of women returning to employment after their first childbirth is significantly associated with their socioeconomic status (Arntz et al. 2017) and the expected career cost of children as indicated by the type of tasks done (Adda et al. 2017). Women employed in jobs primarily involving non-routine cognitive tasks (analytical and interactive) have the highest probability of transitioning to employment after their first childbirth. Conversely, women in occupations characterised by intensive routine tasks, which increasingly become less attractive, exhibit a higher incidence of transitioning to unemployment. In summary, these results suggest that structural changes in the labour market driven by technology and globalization exacerbate employment disparities by placing mothers who do not hold jobs in high demand and are less career-oriented at a disadvantage regarding their employment status.

The remainder of the paper is organized as follows. Section 2 provides details on the data, the task content of work framework, and the empirical strategy. Section 3 presents the model results. Finally, Sect. 4 concludes.

## Data and methods

### Analytical sample

The primary data source for this analysis is the individual-level administrative data obtained from the German Pension Fund. The Pension Fund offers process-induced labour market data, encompassing approximately 90% of the population, with exceptions for certain professional groups such as farmers, lawyers, doctors, and civil servants (AKVS, FDZ-RV (2024)). While administrative data typically offer less detailed information compared to survey data, they compensate with larger sample sizes, particularly advantageous when studying specific sub-populations, as in the case of mothers in this study. Hence, the dataset from the German Pension Fund is more suitable for the analysis presented here than the German Socioeconomic Panel (SOEP), which records only about 4,000 first births-a figure too small for modelling occupational diversity. Although the Sample of Integrated Labour Market Biographies (SIAB) from the Institute of Employment Research (IAB) provides a sufficiently large 2% random sample of the German workforce, the identification of births in that data relies on information about employment interruptions due to entitlement to other compensation by the statutory health insurance provider, conflating maternity leave with long-term sickness and failing to identify birth parity (Mueller et al. 2017). In contrast, data from the German Pension Fund offers precise dates of subsequent births, as well as parental leave periods with monthly precision. Exact identification of births is pivotal for the analysis presented here for two reasons. First, the first birth holds special significance compared to higher-order parities, defining the exact moment of gender divergence in labour market outcomes (Goldin 2021). Second, the second birth is treated as an explicit competing event in the methodology employed (see details in Sect. 2.3). In summary, administrative data from the German Pension Fund represent the only dataset enabling the analysis undertaken in this study (for Germany).

These data encompass labour market information for over 20 million women in Germany since 2011, with data containing occupational codes (AKVS, FDZ-RV (2024)). However, information on childbirth is available only for a 2% random sample (VSKT, FDZ-RV (2024)), significantly reducing the counts. Further restrictions are applied to the analytical sample: only women with German citizenship are included, as migrant women in Germany typically follow different fertility patterns (Milewski et al. 2010) and fertility histories of women with foreign citizenship are incomplete in the data (Kreyenfeld and Mika 2008). Women who died within the observation period are excluded, thereby disregarding death as a source of right censoring. Moreover, only women who experienced their first birth between the beginning of 2012 and the end of 2018 are retained. This time frame allows for a sufficiently extended period to observe women’s potential return to the labour market before the onset of the Covid-19 pandemic. Lastly, only women aged 20 to 45 at their first childbirth are included, and those who gave birth as a result of a multiple fetus pregnancy are excluded. The final sample comprises 63,929 women.

The data are structured for a competing risk analysis (Fine et al. 1999). Observation of women starts one month after they give birth, and the observation period ends either upon the occurrence of the first considered event or when they are right-censored. The three primary events that new mothers transition to are employment, unemployment, and a second birth. Transitions to employment or unemployment can be directly identified from information on the month when a mother concludes maternity leave (lasting 14 weeks in total, with at least 8 weeks taken after the birth) or parental leave and begins paying social contributions or receives unemployment benefits. However, the monthly data available do not allow for distinguishing transitions to full-time versus part-time employment, representing a limitation in understanding women’s labour market mobility in Germany. Information on the month and year when a woman has a second birth is provided in the data. Transitions to inactivity are not analysed as an event due to the challenge in precisely defining the moment when it occurs. Although transitions to self-employment could theoretically be defined based on the type of social contributions self-employed individuals pay to the Pension Fund, such contributions are voluntary, resulting in the identification of only a subset of self-employed individuals with an unknown share. Given that self-employment is relatively uncommon in Germany, particularly among women (OECD 2023), this poses a minor concern. Transitions to inactivity, self-employment, or other infrequent states (e.g., permanent disability) are treated as censored. The histories are documented with monthly precision, and observation of women begins one month after they give birth. There are no overlapping events. Additionally, it is assumed that the second birth can occur at the earliest after eight full months from the first birth.

Table 2 presents the proportions of women who experienced various events following their first childbirth, with the first event assigned to each of them. Approximately 63% of mothers transitioned to employment as the first event after giving birth. Around 19% transitioned to unemployment, 14% transitioned to a second birth, and 4% were censored. Additionally, Fig. 3 illustrates the percentages of experienced events by the birth year of the first child. While the shares remain relatively stable over time, the proportion of mothers returning to employment decreased between 2017 and 2018. Simultaneously, the proportion of censored women increased during that period. This is attributed to the fact that the sample is censored on February 28, 2020 (i.e., before the Covid-19 pandemic), and mothers who gave birth in 2017 or 2018 had “less time” to transition to employment, unemployment, or a second birth compared to mothers in the sample who gave birth between 2012 and 2016. Consequently, their transitions had not yet been observed. Supplementary Fig. 4 presents the duration in months by event. Most women are censored after approximately 20 months, likely those who had their first child around 2017 and had not re-entered the labor market or had a second child yet. Censored women with longer durations may have permanently transitioned to inactivity. On the other hand, the majority of women returning to employment do so after approximately 12 months. After 40 months of inactivity, mothers rarely return to employment. The pattern is more varied for mothers transitioning to unemployment-it occurs either after the first two months of being a mother or after a year. For women having a second child without returning to the labor market between births, the majority give birth to their second child after approximately 24 months from the first birth.

### Task measures

Next, I construct aggregate measures of task content of work using the 2006 Employment Survey of the German Federal Institute for Vocational Education and Training (BiBB) (Hall et al. 2006) and merge them with the individual data from the German Pension Fund by occupational codes.

To describe and quantify changes in labor demand caused by technology and globalization, economists have proposed using a task-based approach (Autor et al. 2003; Acemoglu et al. 2011). This approach posits that occupations consist of various tasks, and the composition of these tasks is altered with changes in labor demand. Tasks differ in complexity, as well as in the level of skills and education needed to perform them. Technology and globalization have reshaped the structure of tasks demanded in the labor market, automating or offshoring some tasks and creating new ones. As a result, they have altered the demand for skills, impacting workers’ labor market opportunities. The literature has proposed five task domains: analytic, involving activities requiring complex analysis of data or concepts, such as programming or conducting statistical analyses; interactive/interpersonal, covering tasks relying on human interactions, such as counseling or negotiating; non-routine manual, encompassing tasks performed in a non-repetitive manner but using one’s hands, such as massaging or hair styling; routine manual, representing tasks done with one’s hands in a constant way, such as cleaning or sorting goods on a factory production line; and routine cognitive, involving activities of a cognitive nature performed in a routine fashion, such as measuring or bookkeeping (Autor et al. 2003; Spitz-Oener 2006; Hardy et al. 2018). These task categories provide a framework for understanding how technological change and globalization impact the demand for different skills in the labor market.

To assess the content of occupations, I employ five measures based on the work of Autor et al. (2003), adapted to the German context by Spitz-Oener (2006) and Rohrbach-Schmidt and Tiemann (2013). These measures are derived using data from the 2006 Employment Survey of the German Federal Institute for Vocational Education and Training (BiBB) (Hall et al. 2006). The BiBB Employment Survey is a cross-sectional survey conducted every 6-7 years since 1979. The choice of the 2006 survey, rather than a later one, ensures an exogenous measurement of task content of work. The survey comprises over 20,000 participants and includes a comprehensive set of questions about the activities performed at work. Respondents indicate whether they frequently, occasionally, or never perform specific activities. I categorize these activities into the five domains using the criterion validation method proposed by Rohrbach-Schmidt and Tiemann (2013). Table 1 presents these activities along with the categories to which they were classified. It’s important to note that the routine cognitive measure is defined by just one task item, *measuring* (see Table 1), making it potentially unreliable. For this reason, I combine the routine cognitive and routine manual measures together into a *routine* measure.

**Table 1:** Availability of activities performed at work and their classification to task categories

	Activity	Task category
1	Organizing	Analytic
2	Researching	Analytic
3	Investigating	Analytic
4	Programming	Analytic
5	Teaching	Interactive
6	Consulting	Interactive
7	Buying	Interactive
8	Promoting	Interactive
9	Repairing	Non-routine manual
10	Caring	Non-routine manual
11	Accommodating	Non-routine manual
12	Protecting	Non-routine manual
13	Measuring	Routine (cognitive)
14	Operating	Routine (manual)
15	Manufacturing	Routine (manual)
16	Storing	Routine (manual)

The *j* task measure can be expressed as:

1 $$\begin{aligned} j\, \text {task measure}_o = \dfrac{\sum _{i=1}^N j\, \text {task measure}_{o,i}}{N} \end{aligned}$$

where

2 $$\begin{aligned} j \text { task measure}_{o,i} = \dfrac{\text {number of items in category}\, j\, \text {performed by}\, i}{\text {total number of items in category} \,j} \end{aligned}$$

and $j\, \in \,\left\{ {{\text{analytic}},{\text{interactive}},{\text{non}} - {\text{routine manual}},{\text{routine}}} \right\}$. Suppose a worker performs organizing and researching. Their analytic task measure would then be 50, as they engage in two activities out of the four classified under the analytic category (see Table 1). Since task measures quantify proportions, they range from 0 to 100. Equation 1, expressed at an occupation level, is a simple average of individual measures (Eq. 2). Occupation-level aggregated task measures are merged with the individual-level dataset constructed from the German Pension Fund Data using 3 digit occupational codes of the German occupational classification (Klassifikation der Berufe 2010). To avoid simultaneity issues, women’s occupations are assigned to one year before their first childbirth. About 25% of women in the analytical sample change occupation in the year of childbirth - this includes also a shift between having an occupation at all and exiting the labour market or vice versa.

The total number of 3 digit occupational codes used to compute the aggregate task measures is equal to 144, 28 of which rely on fewer than 10 individual observations. This can raise a question of whether the scores calculated using such a low number of observations are reliable. An alternative approach would be to use task indices quantified on a 2 digit level, which would include 37 occupations, all relying on at least 32 individual observations. Figure 7 compares the distributions of the number of individual observations used to calculate task measures on a 3 digit and 2 digit level. The number of cases used for the indices on a 3 digit level is clearly skewed towards zero. However, the distributions of 3 digit and 2 digit task measures for mothers in the sample are very similar (Fig. 8) and highly correlated (Table 8). Hence, I use the more detailed 3 digit task measures in the main analysis presented here and conduct a robustness check with 2 digit task measures, which yields very similar findings.

Figure 5 displays unweighted means of task measures by the birth year of the first child. Since the task measures are fixed in time in my setup, any variation over time would result from substantial changes in the composition of occupations where women are employed a year before the first birth. However, no such variations are visible in Fig. 5. The plot also reveals that the analyzed sample exhibits the highest task intensities for the interactive category. This implies that German women were most frequently employed in occupations intense in such tasks within the considered time period, partially aligning with the recent findings of Matysiak et al. (2024), who identified that women in Europe are overrepresented in outward-oriented social tasks.

### Competing risk

I analyze transitions to events as a competing risk problem. This approach was previously used by Arntz et al. (2017) to study post-birth employment transitions of women in Germany in an earlier period than presented here. It considers the possibility that an individual may experience more than one type of event during the follow-up period (e.g., return to employment or transition to second birth) and enables the estimation of the cumulative incidence of each event type while accounting for the occurrence of competing events. The Cumulative Incidence Function (CIF) represents the marginal probability for each competing event. Marginal probability is defined as the probability of subjects who actually developed the event of interest, regardless of whether they were censored or failed from other competing events. By definition, the marginal probability does not assume the independence of competing events, and it is the most popular approach to analyzing competing events data, due to its appealing interpretation.

Fine et al. (1999) proposed a parametric hazards model that allows modelling the CIF with covariates by treating the CIF as a subdistribution function. The subdistribution function is analogous to the Cox proportional hazard model, except it models a hazard function derived from a CIF. The Fine and Gray subdistribution hazard function for event *e* can be expressed as

3 $$\begin{aligned} {\overline{h}}_{e}(t)= \lim _{\Delta \rightarrow 0}\frac{P(t<T<t+\Delta t \, \, \text {and}\, e) \, | \, T > t \, \, \text {or} \, \, (T \le t \, \, \text {and not}\, e)}{\Delta t}. \end{aligned}$$

The above function estimates the hazard rate for event type *e* at time *t* based on the risk set that remains at time *t* after accounting for all previously occurring event types, which includes competing events. The CIF can be computed from the subdistribution hazard as

4 $$\begin{aligned} \text {CIF}_e(t) = 1 - \text {exp}\{-{\overline{H}}_e(t)\} \end{aligned}$$

where ${\overline{H}}_e(t)=\int _0^t {\overline{h}}_e(t) \, dt$ is the cumulative subhazard.

The CIF-based proportional hazard model is then defined as

5 $$\begin{aligned} {\overline{h}}_e (t|x) = {\overline{h}}_{e, 0}(t) \, \text {exp}(x \beta ). \end{aligned}$$

This model satisfies the proportional hazard assumption for the subpopulation hazard being modeled. I estimate the competing risk models using the Stata-core *stcrreg* command.

### Analytical strategy

The task measures are categorised into five equal groups to accommodate cases where the occupation is unknown-missing values constitute the sixth group and are recorded for women who did not work a year before the first birth or if their occupation was not observed in the data. The task measures are included separately in the models. Thus, I run four models, one for each task measure, for each of the three outcomes (employment, unemployment, second birth). The control variables are consistent across all models and include calendar year, the mother’s age, her residence (Bundesland), and education level (low/unknown, middle, high). Since information on occupation, education, and residence is available in the original data with yearly accuracy, all variables are set to one year before the first childbirth (i.e., lagged by one year with respect to the start of the observation period), except for the calendar period, which corresponds to the year of the event. Age is categorised into four groups.

The results of the models presented here should be interpreted solely as correlations for several reasons. First, the administrative data from the German Pension Fund, which relies on information about social contributions and collects limited personal details, lacks the capability to identify marriages or partnerships. Additionally, it provides no additional job characteristics beyond earning points (total gross income centred around the mean and adjusted for inflation). While I can control for some potential confounders such as region (as women might selectively move to regions with better childcare, see Bauernschuster et al. (2015); Mueller et al. (2020)), I cannot include others like partner’s characteristics or women’s labour market history. Second, the issue of selection into occupations following fertility intentions and labour market abilities is an omnipresent problem. Adda et al. (2017) studied women in the vocational track in Germany and demonstrated that this selection occurs as early as the end of primary school, making it practically impossible to circumvent. Third, constraints in data and methodology limit my ability to assess specific mechanisms (such as the income effect) that sort women into different situations post-birth. Although I have information about earning points at my disposal, income can be considered a bad control (Cinelli et al. 2022) because women with higher incomes might face opportunity costs of childbearing and thus selectively transition to employment rather than experiencing a second birth or inactivity. Additionally, there is currently no statistical method available to conduct mediation analysis in a competing risk setting. However, to explore income as a potential mechanism that channels women into various employment transitions after the first birth, I compute Spearman correlations between the task measures and earning points. Finally, global phenomena may simultaneously impact the content of work and the outcomes. Although setting task measures to 2006 partially addresses this, employing instruments in a competing risk setting presents an unsolved methodological challenge. In all regressions, standard errors are clustered at the occupation level. This clustering approach is employed to mitigate the potential impact of measurement error arising from the hierarchical data structure, where task measures are expressed at the occupation level.

## Results

**Fig. 1:**
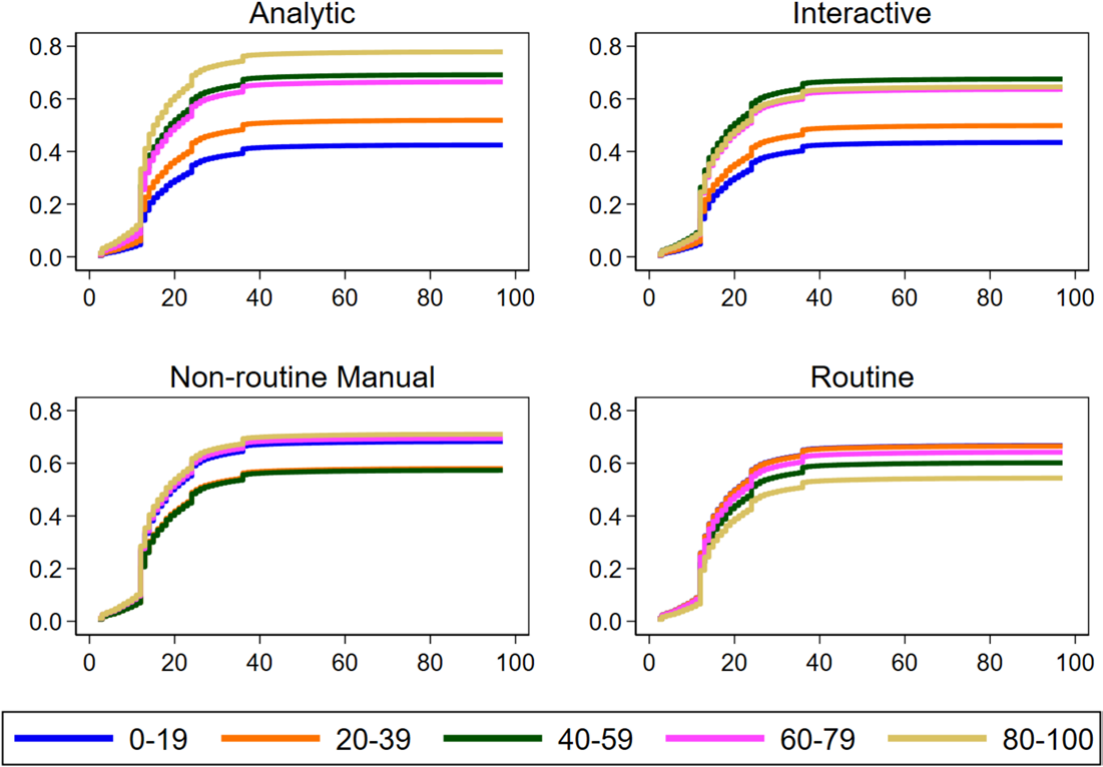
Cumulative Incidence Functions from models with employment set as the main event. Controls include: year of event, age at first childbirth, residence (Bundesland) at first childbirth, education at first childbirth. N = 63,929

**Fig. 2:**
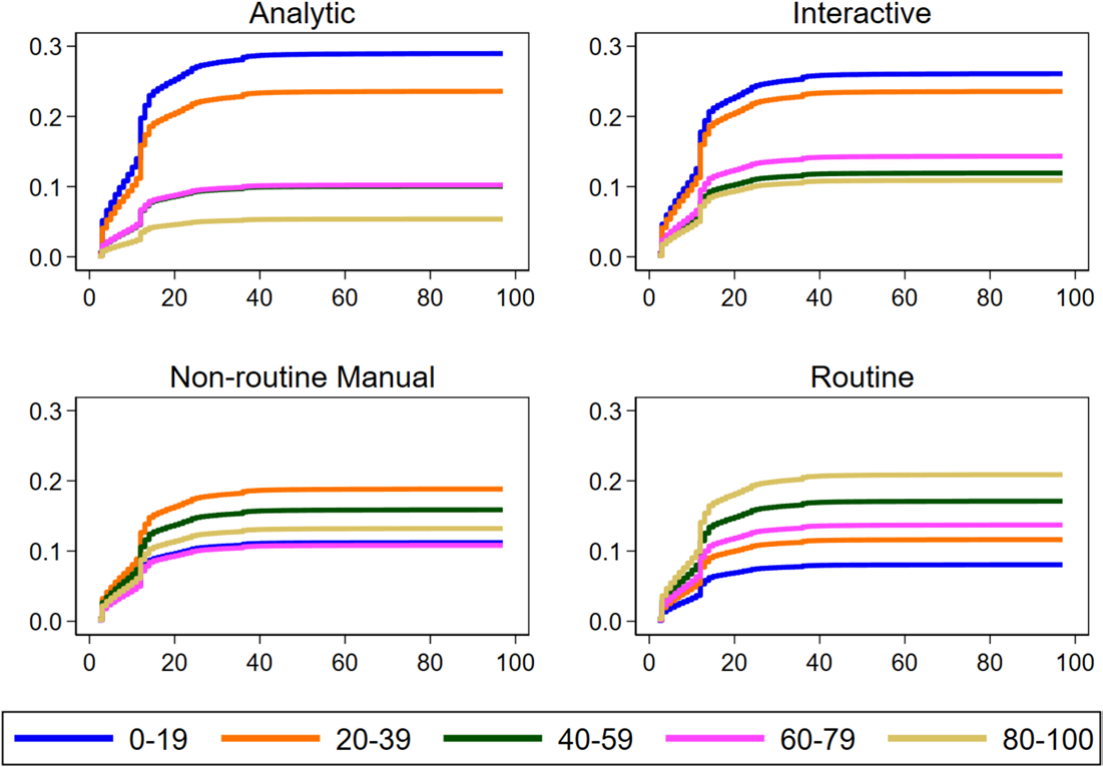
Cumulative Incidence Functions from models with unemployment set as the main event. Controls include: year of event, age at first childbirth, residence (Bundesland) at first childbirth, education at first childbirth. N = 63,929

**Fig. 3:**
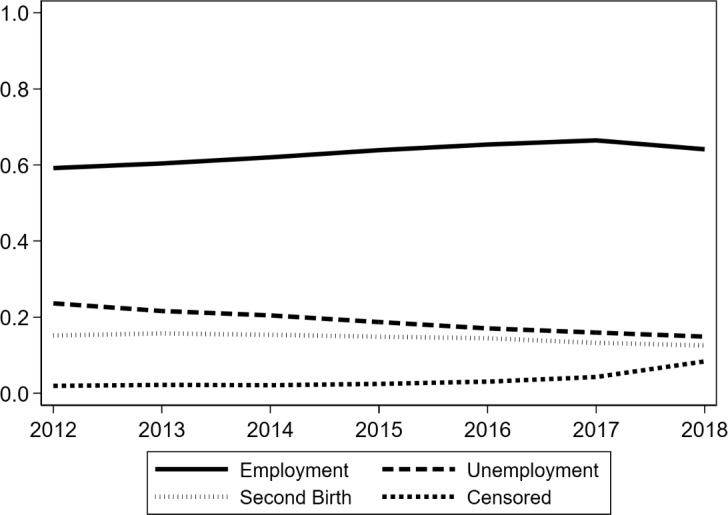
Share of event occurrence by birth year of the first child. N = 63,929

**Fig. 5:**
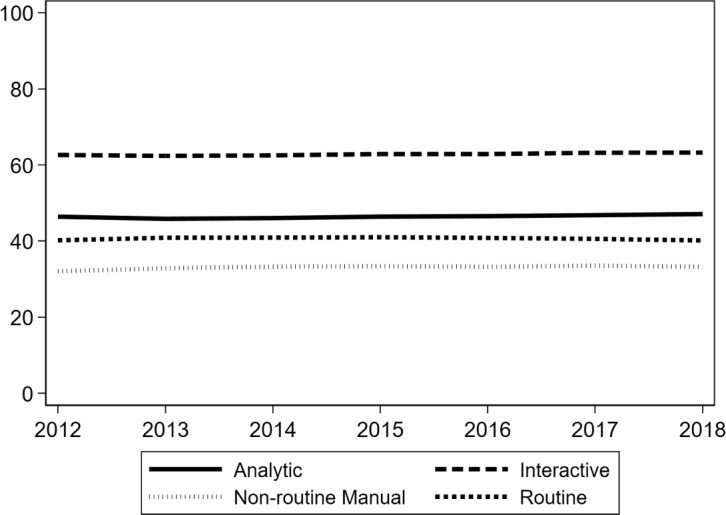
Mean of task measures by birth year of the first child. N = 53,922, i.e. mothers for whom I observe an occupation a year before the first childbirth

**Fig. 6:**
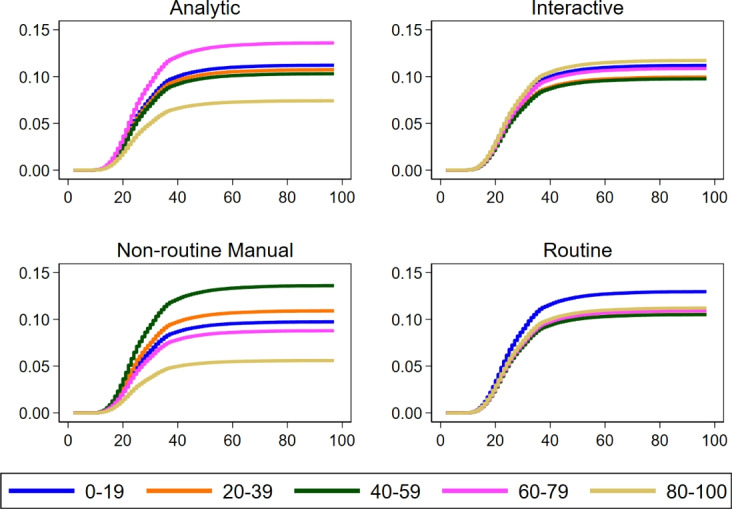
Cumulative Incidence Functions from models with the second birth set as the main event. Controls include: year of event, age at first childbirth, residence (Bundesland) at first childbirth, education at first childbirth. N = 63,929

**Fig. 7:**
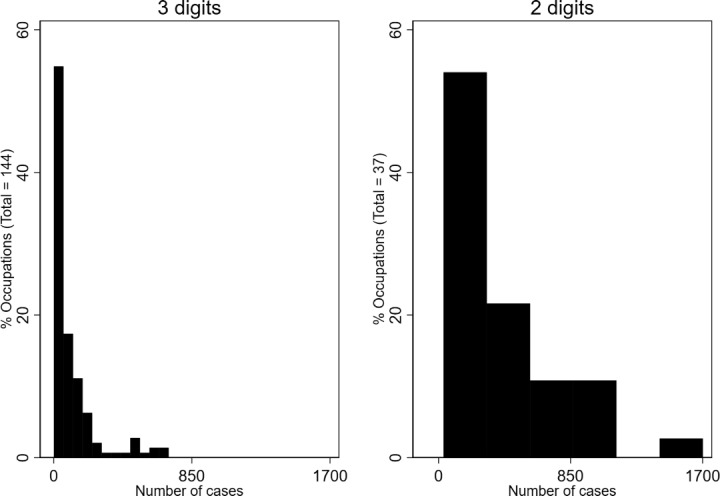
Distirbution of the number of observations in the 2006 BiBB Employment survey used to compute task measures on a 3 digit and 2 digit occupation levels

**Fig. 8:**
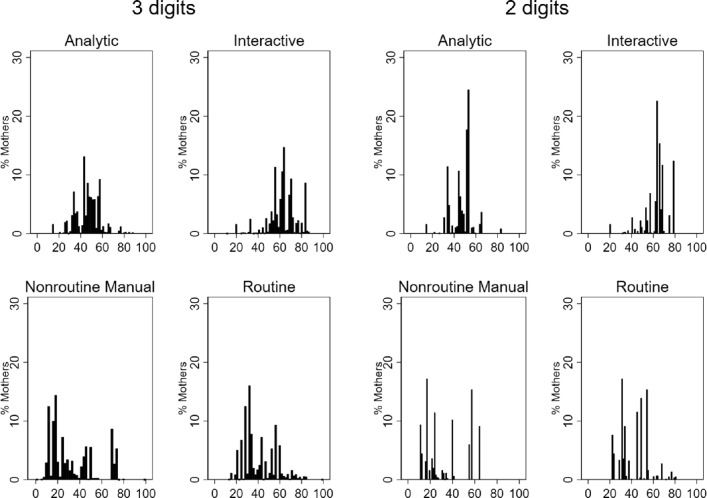
Distribution of task measures for mothers in the sample, comparison of the measures calculated on 3 digit vs. 2 digit occupation level. N = 53,922, i.e. mothers for whom I observe an occupation a year before the first childbirth

Figures 1, 2, and 6 present cumulative incidences of employment, unemployment, and second birth by task measure, with full model results available in Tables 4, 5, 6, and 7. Notably, women with the highest analytic task measure (between 80 and 100) are the most likely to transition to employment after the first birth. Similarly, women with jobs intensive in interactive and non-routine manual tasks also exhibit high cumulative incidences of transitioning to employment, albeit slightly smaller than those with highly analytic jobs. In contrast, women with jobs intense in routine tasks are less likely to transition to employment.

Figure 2 illustrates cumulative incidences of unemployment and shows that women with routine jobs are disproportionately likely to be unemployed after becoming mothers. Correspondingly, women with low analytic and interactive task intensities are also the most likely to transition to unemployment. This transition happens either right after the maternity leave (after 2–3 months) or after the parental leave (after 12 months). These patterns align with economic literature highlighting the labour-replacing consequences of automation and globalization, particularly in routine tasks (e.g., Autor et al. 2003; Hardy et al. 2018). Even if women are guaranteed to return to their job after the maternity/parental leave, they might voluntarily enter inactivity or unemployment, as routine jobs become less attractive. It is also in line with the work of Adda et al. (2017), who showed that family-oriented women, who are overall the most likely to withdraw from the labour market after they become mothers, sorted themselves into routine occupations in Germany.

Additionally, Fig. 6 demonstrates that women with the highest cumulative incidence of a second birth are those with high analytic and low routine measures. Notably, women in the top analytic category record the lowest cumulative incidence of a second birth among all mothers in the sample.

Furthermore, Table 3 presents correlations of continuous task measures with earning points for mothers in the sample. These correlations, given the difference in measurement levels (individual level for earning points and occupation level for task measures), are naturally lower. However, distinct differences between task measures emerge, with the analytic measure showing the highest positive correlation with earnings. On the other hand, the interactive measure exhibits a small positive correlation with earnings, while the two other measures are negatively correlated. This aligns with previous economic research indicating a steady decline in demand for certain types of tasks (Autor et al. 2003; Spitz-Oener 2006; Hardy et al. 2018), as well as wage differentials between task types (Matysiak et al. 2024). These correlations may help explain why women with highly analytic jobs, facing high opportunity costs, are most likely to transition to employment after their first child.

Finally, Tables 9, 10, 11, and 12 show the results of the robustness check in which the task measures are calculated and merged on a 2 digit occupational level. These findings do not differ substantially from the main 3 digit specification presented here.

## Discussion

Technology and globalization have brought about unprecedented changes in the world of work. These transformations have led to a significant polarization of opportunities, particularly between workers with cognitive skills and those with routine/manual skills and occupations. Simultaneously, extensive research on the career impact of childbearing has highlighted socioeconomic disparities in women’s labour market outcomes following the birth of their first child. This study aims to integrate these two strands of literature by investigating how the task content of women’s work, indicative of their long-term labour market situation and their positioning on the career-family continuum, influences their employment transitions after their first childbirth. The study is situated in Germany, a conservative welfare state experiencing the labour-replacing effects of automation and import competition in certain sectors, along with a high demand for cognitive labour in others.

The results of my analysis align with prior research on the European labour market, indicating that women are predominantly employed in jobs characterized by high levels of interactive tasks (Matysiak et al. 2024). This trend persists when focusing specifically on mothers. Additionally, I identified task disparities in the employment transitions of new mothers. Women in jobs involving analytic and interactive tasks were more likely to transition to employment, while those in routine jobs were more prone to moving into unemployment. However, it remains unclear whether this pattern arises from shifting task demands (and voluntary unemployment as a results of diminishing quality and attractiveness of routine jobs), differences in women’s career-family orientations, changing gender norms, or depreciating human capital. Several limitations affect this research. First, due to data constraints, I could not control for potentially relevant confounders such as partnership status. Second, the issue of selection into occupations based on family orientation was pervasive. Third, I was unable to differentiate between full-time and part-time employment or explore specific underlying mechanisms.

Despite these limitations, this study represents the first attempt to investigate the connection between structural labour market changes, the career impact of children, and mothers’ employment transitions in a contemporary context. While a few studies have examined the influence of labour market changes driven by technology and globalization on female employment and careers (Black and Spitz-Oener 2010; Adda et al. 2017; Brussevich et al. 2019; Matysiak et al. 2024), the aspect of maternity has received relatively little attention in this regard. Given the significant automation witnessed through the adoption of industrial robots and AI, along with increasing trade competition and growing economic inequalities in Europe (Piketty and Goldhammer 2014), understanding the intersection of these phenomena is crucial for comprehending their implications for social inequality.

## Figures and Tables

**Fig. 4:**
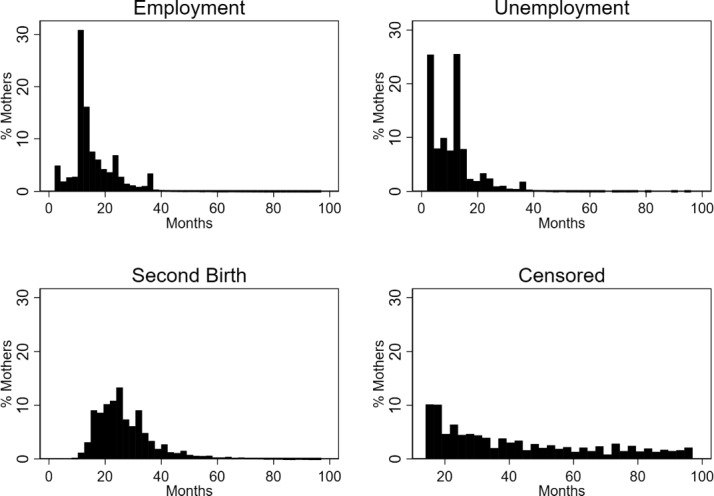
Duration by event in months. N = 63,929

## Data Availability

This paper utilized the AKVS and VSKT administrative datasets, accessible at the Research Data Centre of the German Pension Fund (FDZ-RV). The BiBB Employment Survey 2006 Scientific Use File is obtainable from the Federal Institute for Vocational Education and Training at https://doi.org/10.7803/501.06.1.1.30. Codes employed to generate the analyses presented in this paper can be accessed at https://github.com/LabFam/Bogusz_2024_JLMR.

## Appendix

### Appendix A

See Tables 2, 3, 4, 5, 6, 7 and Figs. 3, 4, 5, 6.

**Table 2:** Share of event occurrence for mothers in the sample

	Event	Count	Share (%)
1	Employment	40,371	63.15
2	Unemployment	12,053	18.85
3	Second birth	9,265	14,49
4	Censored	2.240	3,50
	Total	63,929	100

**Table 3:** Correlation of task measures with earning points for mothers in the sample

Task Measure	Correlation with earning points
Analytic	0.3508
Interactive	0.0731
Non-routine manual	− 0.1720
Routine	−0.1974

Earning points are calculated by centering the total gross income around the mean and they are adjusted for inflation (https://www.gesetze-im-internet.de/sgb_6/anlage_1.html)

**Table 4:** Full model results: analytic task measure, 3 digits

	(1)	(2)	(3)
	Employment	Unemployment	Second birth
Task measure: unknown	1.124 ^***^ (0.018)	0.699 ^***^ (0.010)	1.332 ^***^ (0.023)
Task measure: 0–20	1.000 (.)	1.000 (.)	1.000 (.)
Task measure: 20–40	1.323 ^***^ (0.035)	0.786 ^***^ (0.027)	0.954 (0.039)
Task measure: 40–60	2.127 ^***^ (0.068)	0.310 ^***^ (0.023)	0.914 (0.051)
Task measure: 60–80	1.976 ^***^ (0.212)	0.316 ^***^ (0.042)	1.229 (0.263)
Task measure: 80–100	2.726 ^***^ (0.102)	0.162 ^***^ (0.052)	0.648 ^***^ (0.070)
Age: 20–24	1.000 (.)	1.000 (.)	1.000 (.)
Age: 25–29	1.855 ^***^ (0.110)	0.397 ^***^ (0.013)	1.217 ^**^ (0.120)
Age: 30–34	2.140 ^***^ (0.121)	0.289 ^***^ (0.014)	1.003 (0.152)
Age: 35+	2.305 ^***^ (0.148)	0.316 ^***^ (0.015)	0.540 ^***^ (0.069)
2012–2013	1.000 (.)	1.000 (.)	1.000 (.)
2014–2015	0.770 ^***^ (0.026)	0.739 ^***^ (0.016)	4.685 ^***^ (0.451)
2016–2017	0.742 ^***^ (0.041)	0.584 ^***^ (0.018)	6.157 ^***^ (0.586)
2018–2029	0.764 ^***^ (0.051)	0.450 ^***^ (0.014)	6.606 ^***^ (0.686)
2020	0.301 ^***^ (0.020)	0.150 ^***^ (0.028)	9.737 ^***^ (0.935)
Schleswig-Holstein	1.139 ^***^ (0.040)	0.919 ^*^ (0.047)	0.781 ^***^ (0.067)
Hamburg	1.162 ^***^ (0.033)	0.995 (0.050)	0.673 ^***^ (0.048)
Niedersachsen	1.088 ^***^ (0.022)	0.908 ^**^ (0.034)	0.879 ^***^ (0.038)
Bremen	0.957 (0.062)	1.431 ^***^ (0.096)	0.738 ^**^ (0.098)
Nordrhein-Westfalen	1.000 (.)	1.000 (.)	1.000 (.)
Hessen	1.127 ^***^ (0.033)	0.823 ^***^ (0.046)	0.837 ^***^ (0.050)
Rheinland-Pfalz	1.054 ^**^ (0.026)	0.744 ^***^ (0.032)	1.002 (0.051)
Baden-Wuerttemberg	0.973 (0.022)	0.663 ^***^ (0.024)	1.361 ^***^ (0.044)
Bayern	1.060 ^*^ (0.033)	0.571 ^***^ (0.024)	1.184 ^***^ (0.045)
Saarland	1.177 ^***^ (0.045)	0.980 (0.065)	0.730 ^***^ (0.078)
Berlin	1.266 ^***^ (0.052)	1.364 ^***^ (0.064)	0.342 ^***^ (0.042)
Brandenburg	1.792 ^***^ (0.063)	0.883 ^**^ (0.054)	0.126 ^***^ (0.037)
Mecklenburg-Vorpommern	1.572 ^***^ (0.087)	1.112 (0.096)	0.203 ^***^ (0.043)
Sachsen	1.421 ^***^ (0.049)	0.992 (0.058)	0.260 ^***^ (0.024)
Sachsen-Anhalt	1.531 ^***^ (0.075)	1.098 (0.070)	0.187 ^***^ (0.039)
Thueringen	1.555 ^***^ (0.057)	1.127 ^*^ (0.071)	0.153 ^***^ (0.025)
Education: low/unknown	1.000 (.)	1.000 (.)	1.000 (.)
Education: middle	1.182 ^***^ (0.035)	0.700 ^***^ (0.026)	1.021 (0.038)
Education: high	1.336 ^***^ (0.053)	0.538 ^***^ (0.043)	1.023 (0.093)
Observations	62,325	62,325	62,325

Exponentiated coefficients; Standard errors in parentheses

* $$p<0.10$$, **$$p<0.05$$, *** $$p<0.01$$

**Table 5:** Full model results: interactive task measure, 3 digits

	(1)	(2)	(3)
	Employment	Unemployment	Second birth
Task measure: unknown	1.164 ^***^ (0.028)	0.669 ^***^ (0.016)	1.329 ^***^ (0.024)
Task measure: 0–20	1.000 (.)	1.000 (.)	1.000 (.)
Task measure: 20–40	1.211 ^***^ (0.066)	0.889 ^***^ (0.024)	0.882 ^***^ (0.034)
Task measure: 40–60	1.974 ^***^ (0.083)	0.420 ^***^ (0.051)	0.866 ^**^ (0.053)
Task measure: 60–80	1.774 ^***^ (0.122)	0.512 ^***^ (0.067)	0.969 (0.050)
Task measure: 80–100	1.819 ^***^ (0.145)	0.381 ^***^ (0.017)	1.051 (0.181)
Age: 20–24	1.000 (.)	1.000 (.)	1.000 (.)
Age: 25–29	1.962 ^***^ (0.105)	0.365 ^***^ (0.014)	1.211 ^**^ (0.117)
Age: 30–34	2.318 ^***^ (0.131)	0.251 ^***^ (0.018)	1.000 (0.148)
Age: 35+	2.517 ^***^ (0.166)	0.268 ^***^ (0.021)	0.539 ^***^ (0.067)
2012–2013	1.000 (.)	1.000 (.)	1.000 (.)
2014–2015	0.775 ^***^ (0.026)	0.749 ^***^ (0.017)	4.674 ^***^ (0.451)
2016–2017	0.747 ^***^ (0.040)	0.604 ^***^ (0.019)	6.138 ^***^ (0.581)
2018–2029	0.772 ^***^ (0.050)	0.464 ^***^ (0.016)	6.573 ^***^ (0.679)
2020	0.300 ^***^ (0.019)	0.162 ^***^ (0.033)	9.713 ^***^ (0.916)
Schleswig–Holstein	1.127 ^***^ (0.039)	0.945 (0.049)	0.783 ^***^ (0.068)
Hamburg	1.154 ^***^ (0.033)	0.995 (0.053)	0.674 ^***^ (0.048)
Niedersachsen	1.075 ^***^ (0.023)	0.927 ^**^ (0.035)	0.881 ^***^ (0.038)
Bremen	0.931 (0.062)	1.466 ^***^ (0.094)	0.742 ^**^ (0.098)
Nordrhein-Westfalen	1.000 (.)	1.000 (.)	1.000 (.)
Hessen	1.125 ^***^ (0.033)	0.820 ^***^ (0.046)	0.840 ^***^ (0.050)
Rheinland-Pfalz	1.045 ^*^ (0.026)	0.766 ^***^ (0.034)	1.006 (0.050)
Baden-Wuerttemberg	0.972 (0.022)	0.672 ^***^ (0.023)	1.373 ^***^ (0.042)
Bayern	1.061 ^*^ (0.033)	0.578 ^***^ (0.025)	1.192 ^***^ (0.043)
Saarland	1.151 ^***^ (0.048)	1.000 (0.060)	0.730 ^***^ (0.078)
Berlin	1.249 ^***^ (0.052)	1.373 ^***^ (0.067)	0.344 ^***^ (0.043)
Brandenburg	1.758 ^***^ (0.062)	0.891 ^*^ (0.055)	0.126 ^***^ (0.037)
Mecklenburg–Vorpommern	1.517 ^***^ (0.086)	1.123 (0.102)	0.204 ^***^ (0.044)
Sachsen	1.401 ^***^ (0.047)	1.001 (0.057)	0.263 ^***^ (0.024)
Sachsen–Anhalt	1.490 ^***^ (0.069)	1.123 ^*^ (0.069)	0.188 ^***^ (0.039)
Thueringen	1.537 ^***^ (0.053)	1.131 ^**^ (0.068)	0.154 ^***^ (0.025)
Education: low/unknown	1.000 (.)	1.000 (.)	1.000 (.)
Education: middle	1.268 ^***^ (0.056)	0.611 ^***^ (0.041)	1.006 (0.038)
Education: high	1.525 ^***^ (0.095)	0.386 ^***^ (0.041)	1.047 (0.122)
Observations	62,325	62,325	62,325

Exponentiated coefficients; Standard errors in parentheses

* $$p<0.10$$, ** $$p<0.05$$, *** $$p<0.01$$

**Table 6:** Full model results: non-routine manual task measure, 3 digits

	(1)	(2)	(3)
	Employment	Unemployment	Second birth
Task measure: unknown	0.580 ^***^ (0.046)	1.660 ^***^ (0.290)	1.607 ^***^ (0.080)
Task measure: 0–20	1.000 (.)	1.000 (.)	1.000 (.)
Task measure: 20–40	0.756 ^***^ (0.051)	1.756 ^***^ (0.285)	1.128 ^**^ (0.068)
Task measure: 40–60	0.743 ^***^ (0.055)	1.454 (0.331)	1.427 ^***^ (0.149)
Task measure: 60–80	1.033 (0.077)	0.963 (0.203)	0.897 (0.080)
Task measure: 80–100	1.080 (0.079)	1.192 (0.374)	0.562 ^***^ (0.125)
Age: 20–24	1.000 (.)	1.000 (.)	1.000 (.)
Age: 25–29	1.941 ^***^ (0.109)	0.369 ^***^ (0.014)	1.230 ^**^ (0.116)
Age: 30–34	2.269 ^***^ (0.134)	0.258 ^***^ (0.017)	1.023 (0.148)
Age: 35+	2.435 ^***^ (0.164)	0.280 ^***^ (0.019)	0.554 ^***^ (0.066)
2012–2013	1.000 (.)	1.000 (.)	1.000 (.)
2014–2015	0.772 ^***^ (0.026)	0.751 ^***^ (0.018)	4.685 ^***^ (0.451)
2016–2017	0.747 ^***^ (0.040)	0.598 ^***^ (0.019)	6.127 ^***^ (0.579)
2018–2029	0.772 ^***^ (0.050)	0.457 ^***^ (0.016)	6.565 ^***^ (0.673)
2020	0.304 ^***^ (0.019)	0.158 ^***^ (0.031)	9.572 ^***^ (0.883)
Schleswig–Holstein	1.133 ^***^ (0.040)	0.933 (0.049)	0.776 ^***^ (0.067)
Hamburg	1.161 ^***^ (0.035)	0.992 (0.057)	0.672 ^***^ (0.048)
Niedersachsen	1.091 ^***^ (0.023)	0.915 ^**^ (0.034)	0.875 ^***^ (0.038)
Bremen	0.935 (0.060)	1.472 ^***^ (0.092)	0.750 ^**^ (0.102)
Nordrhein-Westfalen	1.000 (.)	1.000 (.)	1.000 (.)
Hessen	1.127 ^***^ (0.033)	0.823 ^***^ (0.046)	0.839 ^***^ (0.052)
Rheinland–Pfalz	1.053 ^**^ (0.026)	0.749 ^***^ (0.032)	1.003 (0.051)
Baden–Wuerttemberg	0.975 (0.022)	0.666 ^***^ (0.022)	1.363 ^***^ (0.044)
Bayern	1.067 ^**^ (0.034)	0.568 ^***^ (0.022)	1.185 ^***^ (0.045)
Saarland	1.167 ^***^ (0.046)	0.977 (0.064)	0.724 ^***^ (0.077)
Berlin	1.272 ^***^ (0.051)	1.322 ^***^ (0.058)	0.340 ^***^ (0.042)
Brandenburg	1.769 ^***^ (0.063)	0.880 ^**^ (0.051)	0.126 ^***^ (0.037)
Mecklenburg–Vorpommern	1.573 ^***^ (0.088)	1.076 (0.099)	0.199 ^***^ (0.042)
Sachsen	1.427 ^***^ (0.045)	0.975 (0.047)	0.258 ^***^ (0.024)
Sachsen–Anhalt	1.513 ^***^ (0.075)	1.092 (0.070)	0.186 ^***^ (0.039)
Thueringen	1.550 ^***^ (0.055)	1.123 ^*^ (0.072)	0.153 ^***^ (0.025)
Education: low/unknown	1.000 (.)	1.000 (.)	1.000 (.)
Education: middle	1.279 ^***^ (0.057)	0.585 ^***^ (0.037)	1.056 (0.035)
Education: high	1.522 ^***^ (0.097)	0.370 ^***^ (0.046)	1.132 (0.137)
Observations	62,307	62,307	62,307

Exponentiated coefficients; Standard errors in parentheses

* $$p<0.10$$, ** $$p<0.05$$, *** $$p<0.01$$

**Table 7:** Full model results: routine task measure, 3 digits

	(1)	(2)	(3)
	Employment	Unemployment	Second birth
Task measure: unknown	0.614 ^***^ (0.057)	2.338 ^***^ (0.692)	1.135 ^***^ (0.055)
Task measure: 0–20	1.000 (.)	1.000 (.)	1.000 (.)
Task measure: 20–40	0.995 (0.081)	1.473 (0.449)	0.802 ^***^ (0.042)
Task measure: 40–60	0.837 (0.093)	2.236 ^**^ (0.713)	0.800 ^***^ (0.061)
Task measure: 60–80	0.933 (0.092)	1.758 (0.606)	0.833 ^**^ (0.072)
Task measure: 80–100	0.713 ^***^ (0.080)	2.791 ^***^ (0.882)	0.856 ^**^ (0.058)
Age: 20–24	1.000 (.)	1.000 (.)	1.000 (.)
Age: 25–29	1.944 ^***^ (0.106)	0.372 ^***^ (0.016)	1.213 ^**^ (0.119)
Age: 30–34	2.282 ^***^ (0.128)	0.261 ^***^ (0.018)	0.996 (0.150)
Age: 35+	2.468 ^***^ (0.156)	0.282 ^***^ (0.018)	0.535 ^***^ (0.069)
2012–2013	1.000 (.)	1.000 (.)	1.000 (.)
2014–2015	0.772 ^***^ (0.026)	0.751 ^***^ (0.018)	4.681 ^***^ (0.451)
2016–2017	0.745 ^***^ (0.040)	0.602 ^***^ (0.019)	6.159 ^***^ (0.587)
2018–2029	0.769 ^***^ (0.050)	0.462 ^***^ (0.016)	6.601 ^***^ (0.686)
2020	0.299 ^***^ (0.020)	0.162 ^***^ (0.033)	9.775 ^***^ (0.944)
Schleswig-Holstein	1.130 ^***^ (0.039)	0.938 (0.050)	0.783 ^***^ (0.067)
Hamburg	1.162 ^***^ (0.036)	0.995 (0.056)	0.671 ^***^ (0.049)
Niedersachsen	1.082 ^***^ (0.023)	0.922 ^**^ (0.036)	0.880 ^***^ (0.038)
Bremen	0.932 (0.061)	1.462 ^***^ (0.088)	0.741 ^**^ (0.097)
Nordrhein–Westfalen	1.000 (.)	1.000 (.)	1.000 (.)
Hessen	1.128 ^***^ (0.033)	0.826 ^***^ (0.047)	0.837 ^***^ (0.051)
Rheinland–Pfalz	1.047 ^*^ (0.026)	0.759 ^***^ (0.033)	1.008 (0.049)
Baden–Wuerttemberg	0.973 (0.023)	0.672 ^***^ (0.023)	1.367 ^***^ (0.043)
Bayern	1.063 ^*^ (0.034)	0.576 ^***^ (0.024)	1.189 ^***^ (0.043)
Saarland	1.163 ^***^ (0.047)	0.973 (0.062)	0.730 ^***^ (0.078)
Berlin	1.260 ^***^ (0.053)	1.347 ^***^ (0.063)	0.345 ^***^ (0.043)
Brandenburg	1.763 ^***^ (0.063)	0.883 ^**^ (0.051)	0.126 ^***^ (0.037)
Mecklenburg–Vorpommern	1.547 ^***^ (0.088)	1.080 (0.104)	0.203 ^***^ (0.043)
Sachsen	1.413 ^***^ (0.044)	0.987 (0.050)	0.262 ^***^ (0.024)
Sachsen–Anhalt	1.498 ^***^ (0.070)	1.095 (0.070)	0.188 ^***^ (0.039)
Thueringen	1.539 ^***^ (0.052)	1.127 ^*^ (0.069)	0.154 ^***^ (0.025)
Education: low/unknown	1.000 (.)	1.000 (.)	1.000 (.)
Education: middle	1.312 ^***^ (0.066)	0.568 ^***^ (0.046)	1.004 (0.041)
Education: high	1.534 ^***^ (0.103)	0.383 ^***^ (0.046)	1.058 (0.137)
Observations	62,325	62,325	62,325

Exponentiated coefficients; Standard errors in parenthesess

* $$p<0.10$$, ** $$p<0.05$$, *** $$p<0.01$$

### Appendix B: Sensitivity analysis

See Tables 8, 9, 10, 11, 12 and Figs. 7, 8.

**Table 8:** Correlation of continuous task measures calculated on a 3 digit level and 2 digit level for mothers in the sample

Task measure	Correlation 3 digits with 2 digits
Analytic	0.8924
Interactive	0.8692
Non-routine manual	0.9041
Routine	0.8750

N = 53,922, i.e. mothers for whom I observe an occupation a year before the first childbirth

**Table 9:** Full model results: analytic task measure, 2 digits

	(1)	(2)	(3)
	Employment	Unemployment	Second birth
Task measure: unknown	1.137 ^***^ (0.024)	0.691 ^***^ (0.010)	1.327 ^***^ (0.024)
Task measure: 0–20	1.000 (.)	1.000 (.)	1.000 (.)
Task measure: 20–40	1.368 ^***^ (0.022)	0.771 ^***^ (0.019)	0.927 ^***^ (0.024)
Task measure: 40–60	2.034 ^***^ (0.082)	0.352 ^***^ (0.034)	0.937 (0.049)
Task measure: 60–80	1.942 ^***^ (0.258)	0.328 ^***^ (0.053)	1.230 (0.288)
Task measure: 80–100	2.613 ^***^ (0.120)	0.184 ^***^ (0.008)	0.616 ^***^ (0.032)
Age: 20–24	1.000 (.)	1.000 (.)	1.000 (.)
Age: 25–29	1.888 ^***^ (0.124)	0.388 ^***^ (0.017)	1.209 ^*^ (0.126)
Age: 30–34	2.200 ^***^ (0.147)	0.276 ^***^ (0.018)	0.994 (0.158)
Age: 35+	2.377 ^***^ (0.168)	0.299 ^***^ (0.019)	0.536 ^***^ (0.071)
2012–2013	1.000 (.)	1.000 (.)	1.000 (.)
2014–2015	0.770 ^***^ (0.032)	0.744 ^***^ (0.015)	4.683 ^***^ (0.582)
2016–2017	0.743 ^***^ (0.049)	0.593 ^***^ (0.021)	6.157 ^***^ (0.682)
2018–2029	0.765 ^***^ (0.061)	0.457 ^***^ (0.019)	6.602 ^***^ (0.823)
2020	0.299 ^***^ (0.025)	0.155 ^***^ (0.032)	9.765 ^***^ (1.101)
Schleswig–Holstein	1.142 ^***^ (0.047)	0.922 (0.048)	0.782 ^***^ (0.069)
Hamburg	1.163 ^***^ (0.026)	0.981 (0.049)	0.673 ^***^ (0.061)
Niedersachsen	1.084 ^***^ (0.026)	0.916 ^**^ (0.034)	0.880 ^***^ (0.042)
Bremen	0.951 (0.062)	1.449 ^***^ (0.098)	0.738 ^***^ (0.080)
Nordrhein–Westfalen	1.000 (.)	1.000 (.)	1.000 (.)
Hessen	1.126 ^***^ (0.031)	0.820 ^***^ (0.046)	0.837 ^***^ (0.050)
Rheinland–Pfalz	1.052 ^**^ (0.021)	0.742 ^***^ (0.028)	1.004 (0.043)
Baden–Wuerttemberg	0.972 (0.024)	0.667 ^***^ (0.018)	1.364 ^***^ (0.048)
Bayern	1.060 ^*^ (0.036)	0.573 ^***^ (0.020)	1.186 ^***^ (0.046)
Saarland	1.168 ^***^ (0.050)	0.981 (0.078)	0.729 ^**^ (0.093)
Berlin	1.268 ^***^ (0.048)	1.347 ^***^ (0.059)	0.343 ^***^ (0.035)
Brandenburg	1.786 ^***^ (0.062)	0.878 ^**^ (0.051)	0.126 ^***^ (0.037)
Mecklenburg-Vorpommern	1.566 ^***^ (0.109)	1.097 (0.106)	0.203 ^***^ (0.051)
Sachsen	1.415 ^***^ (0.046)	0.994 (0.049)	0.260 ^***^ (0.027)
Sachsen-Anhalt	1.512 ^***^ (0.072)	1.105 (0.068)	0.188 ^***^ (0.036)
Thueringen	1.546 ^***^ (0.050)	1.118 ^*^ (0.072)	0.154 ^***^ (0.029)
Education: low/unknown	1.000 (.)	1.000 (.)	1.000 (.)
Education: middle	1.208 ^***^ (0.047)	0.673 ^***^ (0.026)	1.010 (0.040)
Education: high	1.389 ^***^ (0.069)	0.490 ^***^ (0.041)	1.016 (0.089)
Observations	62,325	62,325	62,325

Exponentiated coefficients; Standard errors in parentheses

* $$p<0.10$$, ** $$p<0.05$$, *** $$p<0.01$$

**Table 10:** Full model results: interactive task measure, 2 digits

	(1)	(2)	(3)
	Employment	Unemployment	Second birth
Task measure: unknown	1.175 ^***^ (0.034)	0.660 ^***^ (0.016)	1.329 ^***^ (0.023)
Task measure: 0–20	1.000 (.)	1.000 (.)	1.000 (.)
Task measure: 20–40	1.388 ^***^ (0.093)	0.859 ^***^ (0.050)	0.918 ^***^ (0.029)
Task measure: 40–60	1.952 ^***^ (0.158)	0.452 ^***^ (0.107)	0.840 ^***^ (0.046)
Task measure: 60–80	1.773 ^***^ (0.148)	0.508 ^***^ (0.080)	0.965 (0.041)
Age: 20–24	1.000 (.)	1.000 (.)	1.000 (.)
Age: 25–29	1.976 ^***^ (0.122)	0.358 ^***^ (0.015)	1.216 ^*^ (0.122)
Age: 30–34	2.338 ^***^ (0.168)	0.246 ^***^ (0.020)	1.004 (0.156)
Age: 35+	2.540 ^***^ (0.198)	0.263 ^***^ (0.024)	0.540 ^***^ (0.068)
2012–2013	1.000 (.)	1.000 (.)	1.000 (.)
2014–2015	0.775 ^***^ (0.032)	0.752 ^***^ (0.017)	4.679 ^***^ (0.584)
2016–2017	0.747 ^***^ (0.048)	0.605 ^***^ (0.023)	6.143 ^***^ (0.678)
2018–2029	0.773 ^***^ (0.061)	0.463 ^***^ (0.020)	6.585 ^***^ (0.822)
2020	0.300 ^***^ (0.025)	0.162 ^***^ (0.035)	9.721 ^***^ (1.079)
Schleswig–Holstein	1.126 ^***^ (0.046)	0.940 (0.053)	0.784 ^***^ (0.069)
Hamburg	1.154 ^***^ (0.028)	0.998 (0.053)	0.673 ^***^ (0.060)
Niedersachsen	1.075 ^***^ (0.027)	0.931 ^*^ (0.036)	0.882 ^***^ (0.042)
Bremen	0.929 (0.065)	1.478 ^***^ (0.102)	0.744 ^***^ (0.078)
Nordrhein-Westfalen	1.000 (.)	1.000 (.)	1.000 (.)
Hessen	1.122 ^***^ (0.031)	0.820 ^***^ (0.046)	0.840 ^***^ (0.050)
Rheinland–Pfalz	1.042 ^**^ (0.021)	0.763 ^***^ (0.031)	1.011 (0.042)
Baden–Wuerttemberg	0.969 (0.023)	0.674 ^***^ (0.016)	1.374 ^***^ (0.047)
Bayern	1.059 ^*^ (0.037)	0.580 ^***^ (0.021)	1.193 ^***^ (0.045)
Saarland	1.158 ^***^ (0.052)	0.987 (0.073)	0.728 ^**^ (0.093)
Berlin	1.256 ^***^ (0.052)	1.355 ^***^ (0.072)	0.344 ^***^ (0.035)
Brandenburg	1.757 ^***^ (0.060)	0.886 ^**^ (0.054)	0.126 ^***^ (0.037)
Mecklenburg-Vorpommern	1.526 ^***^ (0.113)	1.108 (0.118)	0.203 ^***^ (0.051)
Sachsen	1.401 ^***^ (0.049)	1.000 (0.053)	0.262 ^***^ (0.027)
Sachsen-Anhalt	1.477 ^***^ (0.069)	1.130 ^**^ (0.069)	0.188 ^***^ (0.037)
Thueringen	1.538 ^***^ (0.048)	1.123 ^*^ (0.073)	0.154 ^***^ (0.029)
Education: low/unknown	1.000 (.)	1.000 (.)	1.000 (.)
Education: middle	1.289 ^***^ (0.068)	0.582 ^***^ (0.037)	1.012 (0.038)
Education: high	1.555 ^***^ (0.121)	0.359 ^***^ (0.046)	1.071 (0.119)
Observations	62,325	62,325	62,325

Exponentiated coefficients; Standard errors in parentheses

* $$p<0.10$$, ** $$p<0.05$$, *** $$p<0.01$$

**Table 11:** Full model results: non-routine manual task measure, 2 digits

	(1)	(2)	(3)
	Employment	Unemployment	Second birth
Task measure: unknown	0.578 ^***^ (0.048)	1.852 ^***^ (0.343)	1.540 ^***^ (0.058)
Task measure: 0–20	1.000 (.)	1.000 (.)	1.000 (.)
Task measure: 20–40	0.764 ^***^ (0.062)	1.948 ^***^ (0.353)	1.096 (0.081)
Task measure: 40–60	0.832 ^**^ (0.059)	1.447 (0.407)	1.196 ^**^ (0.085)
Task measure: 60–80	0.925 ^***^ (0.027)	1.179 (0.177)	1.019 (0.024)
Age: 20–24	1.000 (.)	1.000 (.)	1.000 (.)
Age: 25–29	1.939 ^***^ (0.120)	0.372 ^***^ (0.017)	1.227 ^**^ (0.122)
Age: 30–34	2.267 ^***^ (0.150)	0.262 ^***^ (0.018)	1.020 (0.154)
Age: 35+	2.434 ^***^ (0.176)	0.285 ^***^ (0.020)	0.552 ^***^ (0.067)
2012–2013	1.000 (.)	1.000 (.)	1.000 (.)
2014–2015	0.772 ^***^ (0.032)	0.750 ^***^ (0.016)	4.682 ^***^ (0.586)
2016–2017	0.747 ^***^ (0.048)	0.599 ^***^ (0.022)	6.140 ^***^ (0.680)
2018–2029	0.771 ^***^ (0.060)	0.459 ^***^ (0.019)	6.586 ^***^ (0.820)
2020	0.300 ^***^ (0.025)	0.160 ^***^ (0.034)	9.713 ^***^ (1.077)
Schleswig–Holstein	1.130 ^***^ (0.044)	0.939 (0.048)	0.780 ^***^ (0.070)
Hamburg	1.157 ^***^ (0.030)	0.991 (0.055)	0.674 ^***^ (0.061)
Niedersachsen	1.084 ^***^ (0.027)	0.917 ^**^ (0.037)	0.878 ^***^ (0.042)
Bremen	0.944 (0.063)	1.438 ^***^ (0.098)	0.741 ^***^ (0.080)
Nordrhein–Westfalen	1.000 (.)	1.000 (.)	1.000 (.)
Hessen	1.125 ^***^ (0.031)	0.826 ^***^ (0.047)	0.839 ^***^ (0.050)
Rheinland–Pfalz	1.051 ^***^ (0.020)	0.748 ^***^ (0.031)	1.007 (0.042)
Baden–Wuerttemberg	0.975 (0.026)	0.667 ^***^ (0.016)	1.371 ^***^ (0.050)
Bayern	1.063 ^*^ (0.038)	0.572 ^***^ (0.019)	1.192 ^***^ (0.045)
Saarland	1.158 ^***^ (0.051)	0.982 (0.077)	0.727 ^**^ (0.092)
Berlin	1.269 ^***^ (0.055)	1.325 ^***^ (0.070)	0.344 ^***^ (0.035)
Brandenburg	1.774 ^***^ (0.064)	0.879 ^**^ (0.048)	0.126 ^***^ (0.037)
Mecklenburg–Vorpommern	1.557 ^***^ (0.113)	1.084 (0.107)	0.201 ^***^ (0.051)
Sachsen	1.426 ^***^ (0.047)	0.971 (0.042)	0.260 ^***^ (0.027)
Sachsen–Anhalt	1.512 ^***^ (0.072)	1.087 (0.064)	0.186 ^***^ (0.036)
Thueringen	1.549 ^***^ (0.049)	1.116 ^*^ (0.074)	0.154 ^***^ (0.029)
Education: low/unknown	1.000 (.)	1.000 (.)	1.000 (.)
Education: middle	1.303 ^***^ (0.071)	0.582 ^***^ (0.045)	1.011 (0.040)
Education: high	1.562 ^***^ (0.111)	0.353 ^***^ (0.042)	1.095 (0.129)
Observations	62,325	62,325	62,325

Exponentiated coefficients; Standard errors in parentheses * $p<0.10$, ** $p<0.05$, *** $p<0.01$

**Table 12:** Full model results: routine task measure, 2 digits

	(1)	(2)	(3)
	Employment	Unemployment	Second birth
Task measure: unknown	0.959 (0.048)	0.692 ^***^ (0.035)	1.379 ^***^ (0.043)
Task measure: 0–20	1.000 (.)	1.000 (.)	1.000 (.)
Task measure: 20–40	1.606 ^***^ (0.070)	0.409 ^***^ (0.054)	0.933 (0.042)
Task measure: 40–60	1.325 ^***^ (0.107)	0.630 ^***^ (0.113)	1.012 (0.058)
Task measure: 60–80	1.346 ^***^	0.649 ^***^	0.971
	(0.114)	(0.093)	(0.073)
Task measure: 80–100	1.000 (.)	1.000 (.)	1.000 (.)
Age: 20–-24	1.000 (.)	1.000 (.)	1.000 (.)
Age: 25–29	1.941 ^***^ (0.116)	0.370 ^***^ (0.018)	1.225 ^**^ (0.120)
Age: 30–34	2.277 ^***^ (0.145)	0.259 ^***^ (0.019)	1.012 (0.154)
Age: 35+	2.456 ^***^ (0.172)	0.282 ^***^ (0.020)	0.545 ^***^ (0.068)
2012–2013	1.000 (.)	1.000 (.)	1.000 (.)
2014–2015	0.773 ^***^ (0.032)	0.750 ^***^ (0.016)	4.680 ^***^ (0.583)
2016–2017	0.746 ^***^ (0.048)	0.599 ^***^ (0.022)	6.146 ^***^ (0.679)
2018–2029	0.770 ^***^ (0.060)	0.460 ^***^ (0.019)	6.591 ^***^ (0.819)
2020	0.299 ^***^ (0.025)	0.161 ^***^ (0.035)	9.742 ^***^ (1.081)
Schleswig–Holstein	1.130 ^***^ (0.045)	0.938 (0.048)	0.782 ^***^ (0.069)
Hamburg	1.161 ^***^ (0.030)	0.995 (0.055)	0.672 ^***^ (0.061)
Niedersachsen	1.084 ^***^ (0.028)	0.920 ^**^ (0.038)	0.880 ^***^ (0.041)
Bremen	0.935 (0.063)	1.458 ^***^ (0.093)	0.741 ^***^ (0.077)
Nordrhein–Westfalen	1.000 (.)	1.000 (.)	1.000 (.)
Hessen	1.127 ^***^ (0.032)	0.825 ^***^ (0.048)	0.838 ^***^ (0.050)
Rheinland–Pfalz	1.047 ^**^ (0.020)	0.755 ^***^ (0.030)	1.008 (0.042)
Baden–Wuerttemberg	0.975 (0.026)	0.671 ^***^ (0.015)	1.368 ^***^ (0.047)
Bayern	1.064 ^*^ (0.038)	0.574 ^***^ (0.020)	1.190 ^***^ (0.045)
Saarland	1.157 ^***^ (0.051)	0.984 (0.073)	0.729 ^**^ (0.092)
Berlin	1.265 ^***^ (0.051)	1.335 ^***^ (0.066)	0.345 ^***^ (0.035)
Brandenburg	1.768 ^***^ (0.063)	0.877 ^**^ (0.049)	0.126 ^***^ (0.037)
Mecklenburg–Vorpommern	1.555 ^***^ (0.114)	1.067 (0.114)	0.202 ^***^ (0.052)
Sachsen	1.422 ^***^ (0.045)	0.972 (0.042)	0.261 ^***^ (0.028)
Sachsen–Anhalt	1.509 ^***^ (0.072)	1.084 (0.065)	0.187 ^***^ (0.037)
Thueringen	1.544 ^***^ (0.049)	1.119 ^*^ (0.072)	0.154 ^***^ (0.029)
Education: low/unknown	1.000 (.)	1.000 (.)	1.000 (.)
Education: middle	1.309 ^***^ (0.072)	0.568 ^***^ (0.045)	1.012 (0.038)
Education: high	1.512 ^***^ (0.108)	0.389 ^***^ (0.049)	1.089 (0.132)
Observations	62,325	62,325	62,325

Exponentiated coefficients; Standard eAPPENDIXrrors in parentheses

* $$p<0.10$$, ** $$p<0.05$$, *** $$p<0.01$$
